# Crystal structures of two polymorphs for *fac*-bromido­tricarbon­yl[4-(4-meth­oxy­phen­yl)-2-(pyridin-2-yl)thia­zole-κ^2^*N*,*N*′]rhenium(I)

**DOI:** 10.1107/S2056989024010727

**Published:** 2024-11-08

**Authors:** Yuki Matsuda, Ryota Nakamura, Yoshiki Ozawa, Masaaki Abe

**Affiliations:** a Graduate School of Science University of Hyogo, 3-2-1 Koto Kamigori-cho Ako-gun Hyogo 678-1297 Japan; Tokyo University of Science, Japan

**Keywords:** crystal structure, polymorph, rhenium(I) complex, chelating ligand, hydrogen bond, photoluminescence

## Abstract

Crystallization of the title compound, *fac*-[ReBr(ppt-OMe)(CO)_3_] (ppt-OMe = C_15_H_12_N_2_OS), from CH_2_Cl_2_/*n*-pentane (1:5 *v*/*v*) at room temperature gave two polymorphs, which crystallize in monoclinic (*P*2_1_/*c*; α form) and ortho­rhom­bic (*Pna*2_1_; β form) space groups. The Re^I^ complex mol­ecules in either polymorph adopt a six-coordinate octa­hedral geometry with three *facially*-oriented carbonyl ligands, one bromido ligand, and two nitro­gen atoms from one chelating ligand ppt-OMe. In the crystal, both polymorph α and β form di-periodic sheet-like architectures supported by multiple hydrogen bonds. In polymorph α, two types of hydrogen bonds (C—H⋯O) are found while, in polymorph β, four types of hydrogen bonds (C—H⋯O and C—H⋯Br) exist.

## Abstract 2

Crystallization of the title compound from CH_2_Cl_2_/*n*-pentane (1:5 *v*/*v*) at room temperature gave two polymorphs, which crystallize in monoclinic (*P*2_1_/*c*; α form) and ortho­rhom­bic (*Pna*2_1_; β form) space groups. The Re^I^ complex mol­ecules in either polymorph adopt a six-coordinate octa­hedral geometry with three *facially*-oriented carbonyl ligands, one bromido ligand, and two nitro­gen atoms from one chelating ligand ppt-OMe. In the crystal, both polymorph α and β form di-periodic sheet-like architectures supported by multiple hydrogen bonds.

## 1. Chemical context

Octa­hedral six-coordinate *fac*-tri(carbon­yl)halogenorhenium(I) complexes formulated as *fac*-[Re^I^(CO)_3_*X*(*N*^*N*)] (*X* = halogeno ligand, *N*^*N* = bidentate ligand with two N donor atoms such as 2,2′-bi­pyridine) constitute a remarkable class of transition-metal complexes, which have been intensively studied for some decades owing to their enormous inter­est in the versatile fields of science such as synthesis, photo-physics and chemistry (Stout *et al.*, 2020 ▸; Ioachim *et al.*, 2006 ▸), metallo­supra­molecular chemistry (Dinolfo *et al.*, 2004 ▸), catalysis (Talukdar *et al.*, 2020 ▸; Matlachowski *et al.*, 2015 ▸), and biological/medical science (Lo *et al.*, 2006 ▸). Chemical modulations of monodentate halogeno ligands with *X* = F, Cl, and Br and bidentate chelating ligands allow the physicochemical properties of rhenium(I) complexes to be largely and finely tuned in intentional directions (Auvray *et al.*, 2021 ▸; Saldías *et al.*, 2019 ▸). Among many derivatives so far explored, 2,2′-bi­pyridine (Kia & Safari, 2016 ▸) and 1,10-phenanthroline (Záliš *et al.*, 2011 ▸) have been structurally characterized. To further develop the synthetic methodology to tune the nature of *fac*-[Re^I^(CO)_3_*X*(*N*^*N*)] complexes, complexation with unsymmetrical bidentate *N*^*N* ligands may provide an additional approach to be exploited, but the examples are still rare to date.

Organic compounds with a 2-(pyridie-2-yl)thia­zole backbone have been synthesized and structurally identified (WAYSAU: Puji Pamungkas *et al.*, 2022 ▸; ITOSAO: Puji Pamungkas *et al.*, 2021 ▸; HUQSOD: Yamaguchi *et al.*, 2015 ▸).

In our ongoing effort to develop transition-metal complexes using 2-(pyridin-2-yl)thia­zole derivatives as new unsymmetrical *N*^*N-*chelating ligands, we herein report the synthesis and structural determination of compound (I), a *fac*-tri(carbon­yl)bromido­rhenium(I) complex bearing 4-(4-meth­oxy­phen­yl)-2-(pyridin-2-yl)thia­zole, hereafter abbreviated as ppt-OMe.
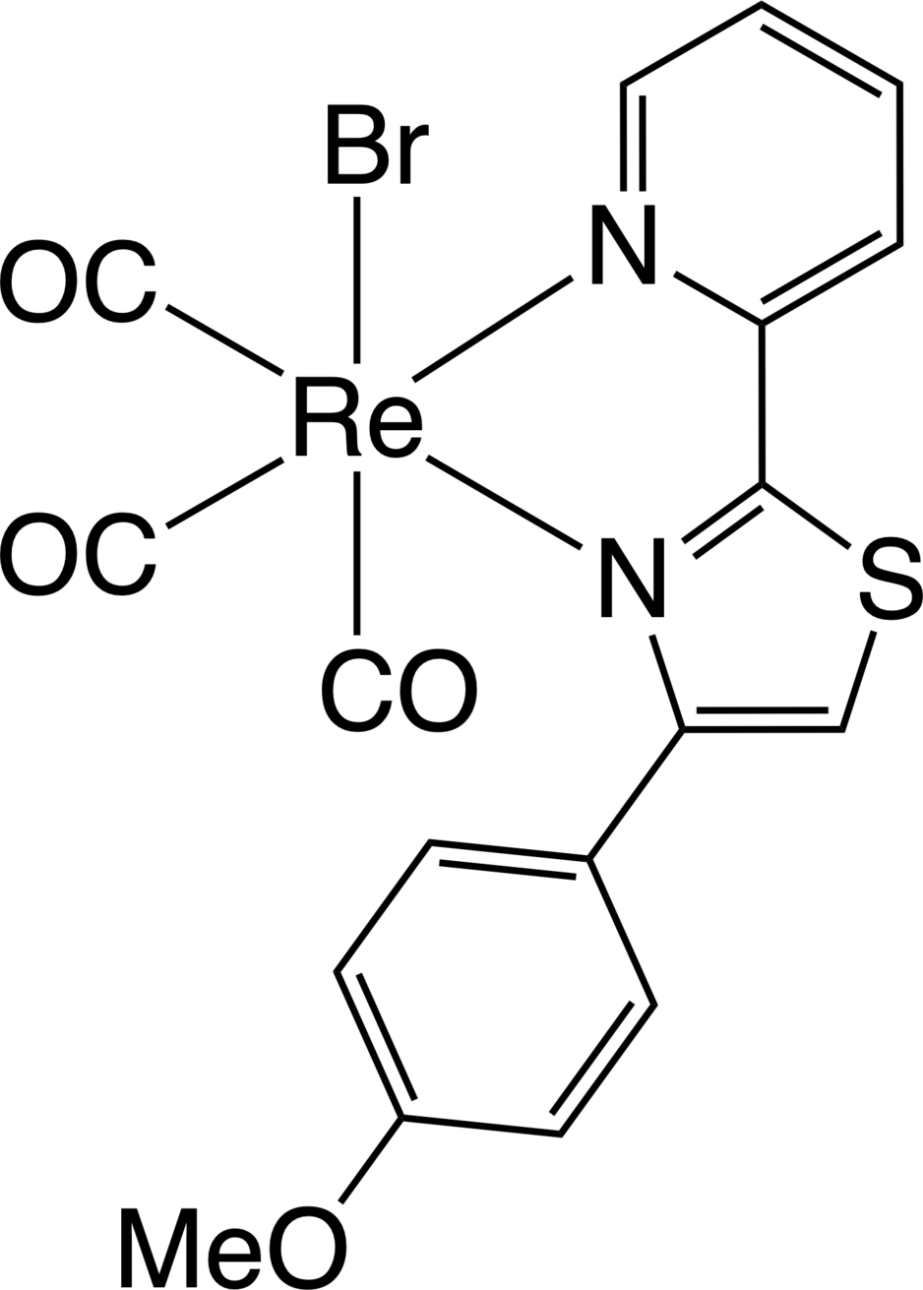


## 2. Structural commentary

**Figure 1:**
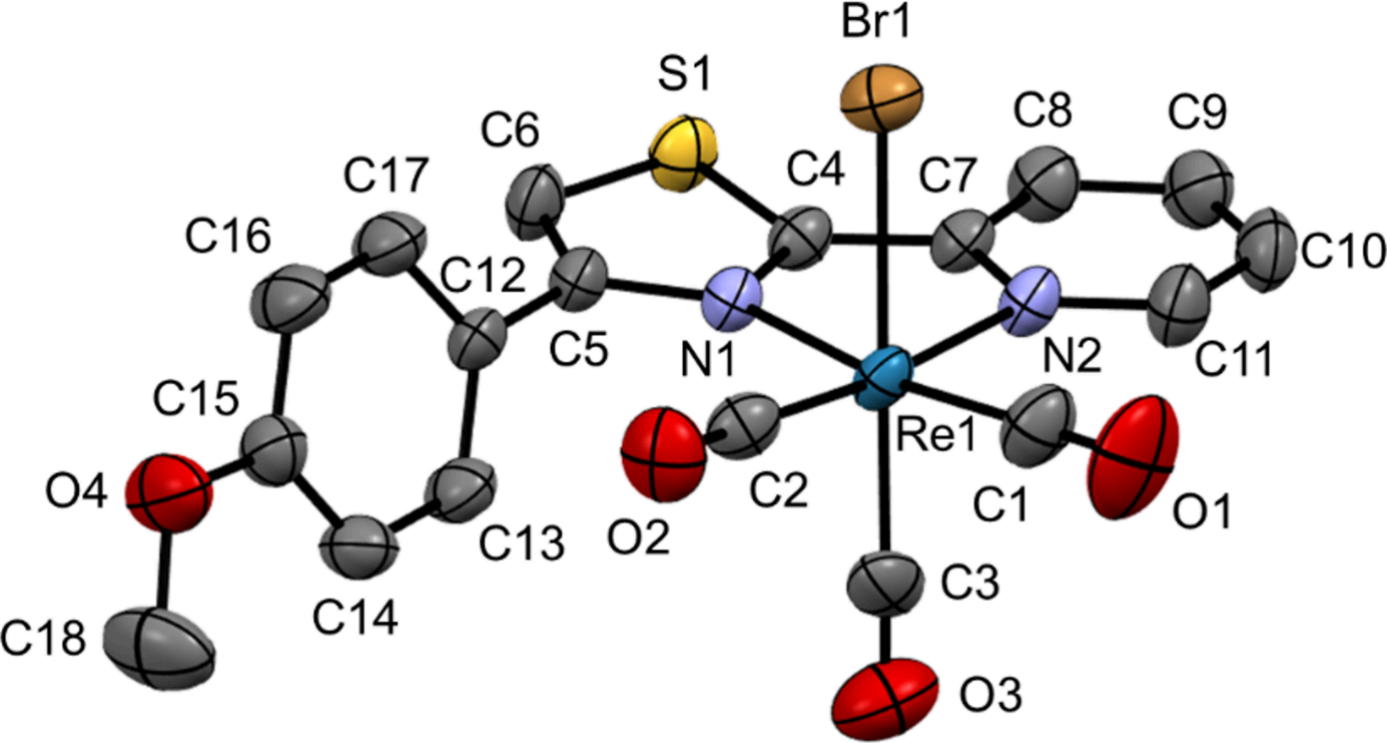
Mol­ecular structure with the atomic labeling scheme for polymorph α of (I) at 296 K, showing displacement ellipsoids at the 50% probability level. Hydrogen atoms are omitted for clarity.

Crystallization of (I) from CH_2_Cl_2_/*n*-pentane (1/5, *v*/*v*) gave two polymorphs, α and β, which differed in the color and shape of the crystals (see details in the *Experimental* section). Polymorph α, bright yellowish orange and rhomboid in shape, crystallizes in the monoclinic space group *P*2_1_/*c*, while polymorph β, vivid orange, pillar shaped, crystallizes in the ortho­rhom­bic space group *Pna*2_1_. The mol­ecular structure of (I) in polymorph α is shown in Fig. 1 ▸. The rhenium(I) center is coordinated by three carbon atoms (C1–C3) from *facially*-oriented carbonyl ligands, one bromido ligand (Br1), and two nitro­gen atoms (N1 and N2) from the chelating ppt-OMe ligand to complete a six-coordinate octa­hedral geometry. The bond lengths and angles around the rhenium center (Re1) are listed in Table 1 ▸. The Re—C bond lengths range between 1.903 (5) and 1.950 (5) Å. The ppt-OMe ligand chelates the rhenium(I) center unsymmetrically with Re1—N1 (Th group) and Re1—N2 (Py group) bond lengths of 2.198 (3) and 2.193 (3) Å, respectively. In the chelating ppt-OMe ligand, the mean planes of the Th and Py rings are almost co-planar, but the Th and phenyl (Ph) rings are twisted, the N1—C5—C12—C17 torsion angle being 118.0 (4)°.

**Figure 2:**
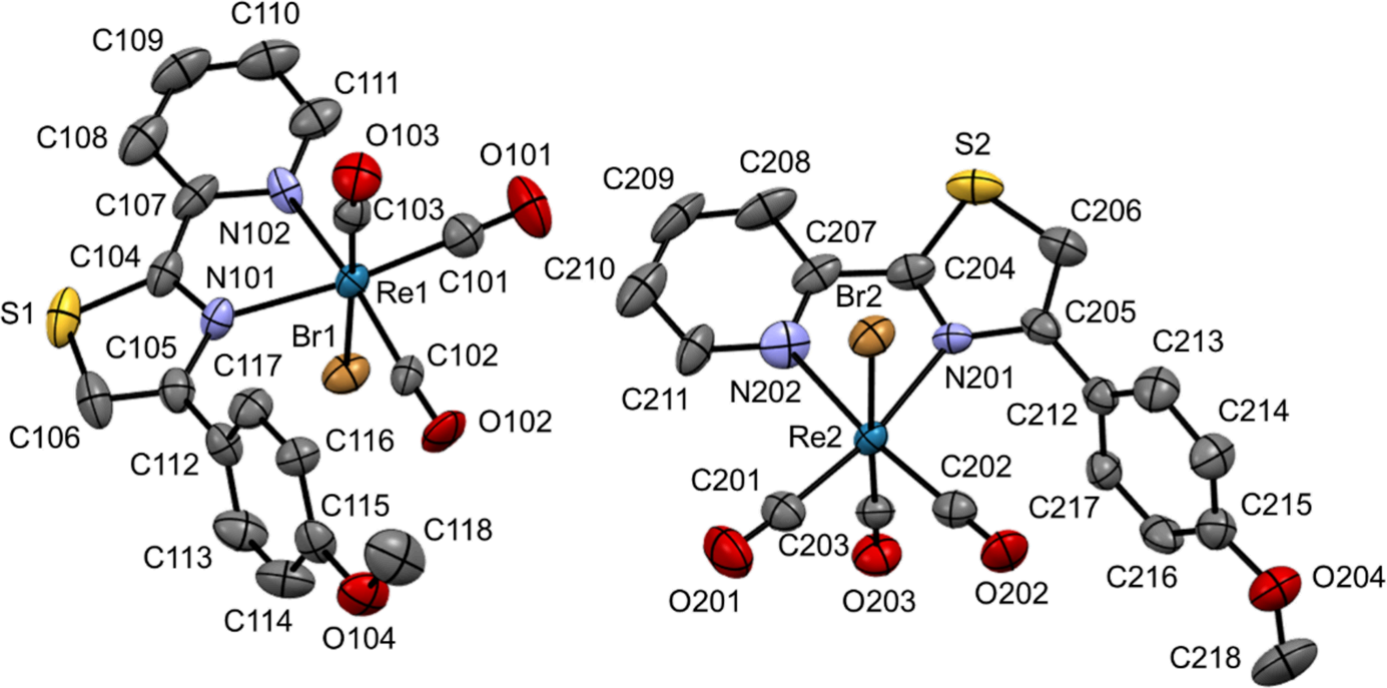
Mol­ecular structures of independent mol­ecules *A* (left) and *B* (right) in polymorph β of (I) at 296 K with the atomic labeling scheme, showing displacement ellipsoids at the 50% probability level. Hydrogen atoms are omitted for clarity.

Polymorph β contains two crystallographically independent mol­ecules, *A* and *B*, in the asymmetric unit (Fig. 2 ▸). Bond lengths around the rhenium centers are listed in Table 2 ▸. As seen in the mol­ecular structure for polymorph α, the complex mol­ecules in polymorph β also adopt a six-coordinate octa­hedral geometry with a {C_3_BrN_2_} donor set. The unsymmet­rical complexation nature of the two nitro­gen donors of ppt-OMe towards the rhenium(I) center is more evident for polymorph β than α in a comparison of the Re—N (Th or Py) bond lengths. The Re—N (Th) bonds are longer than the Re—N (Py) bonds with Re1—N101 (Th) = 2.186 (7) and Re1—N102 (Py) = 2.105 (7) Å for mol­ecule *A* and Re2—N201 (Th) = 2.185 (7) and Re2—N202 (Py) = 2.157 (8) Å for mol­ecule *B*. The Re—C bond lengths range between 1.909 (10) and 1.932 (8) Å. The N101—C105—C112—C117 torsion angle is 61.0 (10)° in mol­ecule *A*, while N201—C205—C212—C217 in mol­ecule *B* is 61.1 (10)°. These angles are almost identical to each other.

## 3. Supra­molecular features

**Figure 3:**
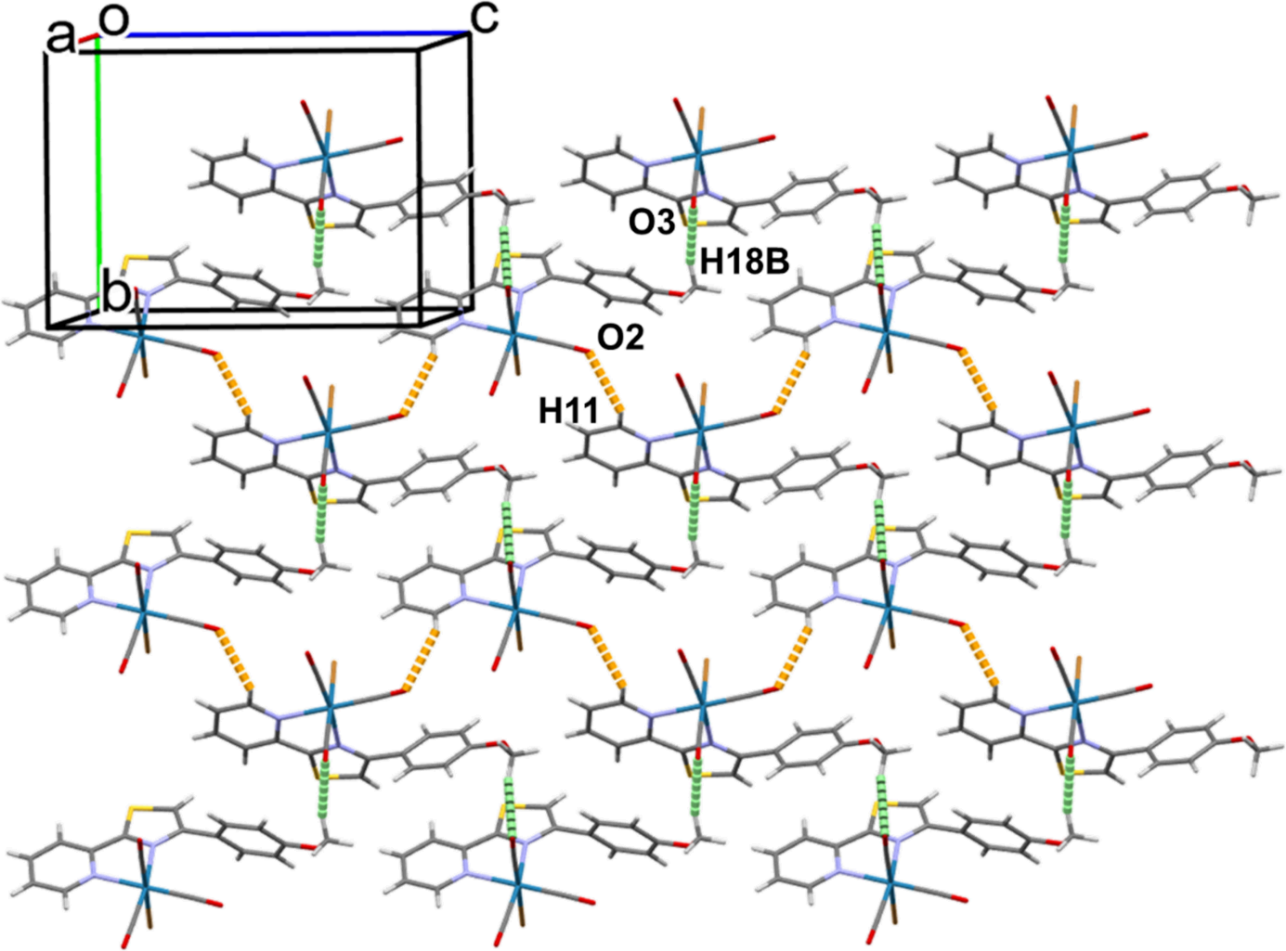
Packing diagram for polymorph α. Hydrogen bonds C18—H18*B*(meth­oxy)⋯O3(carbon­yl) and C11—H11(py)⋯O2(carbon­yl) are shown as dotted lines in green and orange, respectively. Color codes: Re (blue); Br (brown); S (yellow); O (red); N (light blue); C (gray); H (light gray).

Packing diagrams of polymorphs α and β are shown in Figs. 3 ▸ and 4 ▸, respectively. For both polymorphs, hydrogen bonds play an important role in the non-covalent supra­molecular architectures.

In polymorph α (Fig. 3 ▸), two types of hydrogen bonds, C11—H11(Py)⋯O2 (carbon­yl) and C18—H18*B*(meth­oxy)⋯O3 (carbon­yl) (Table 3 ▸), lead to the formation of a di-periodic sheet-like network in the *bc* plane.

**Figure 4:**
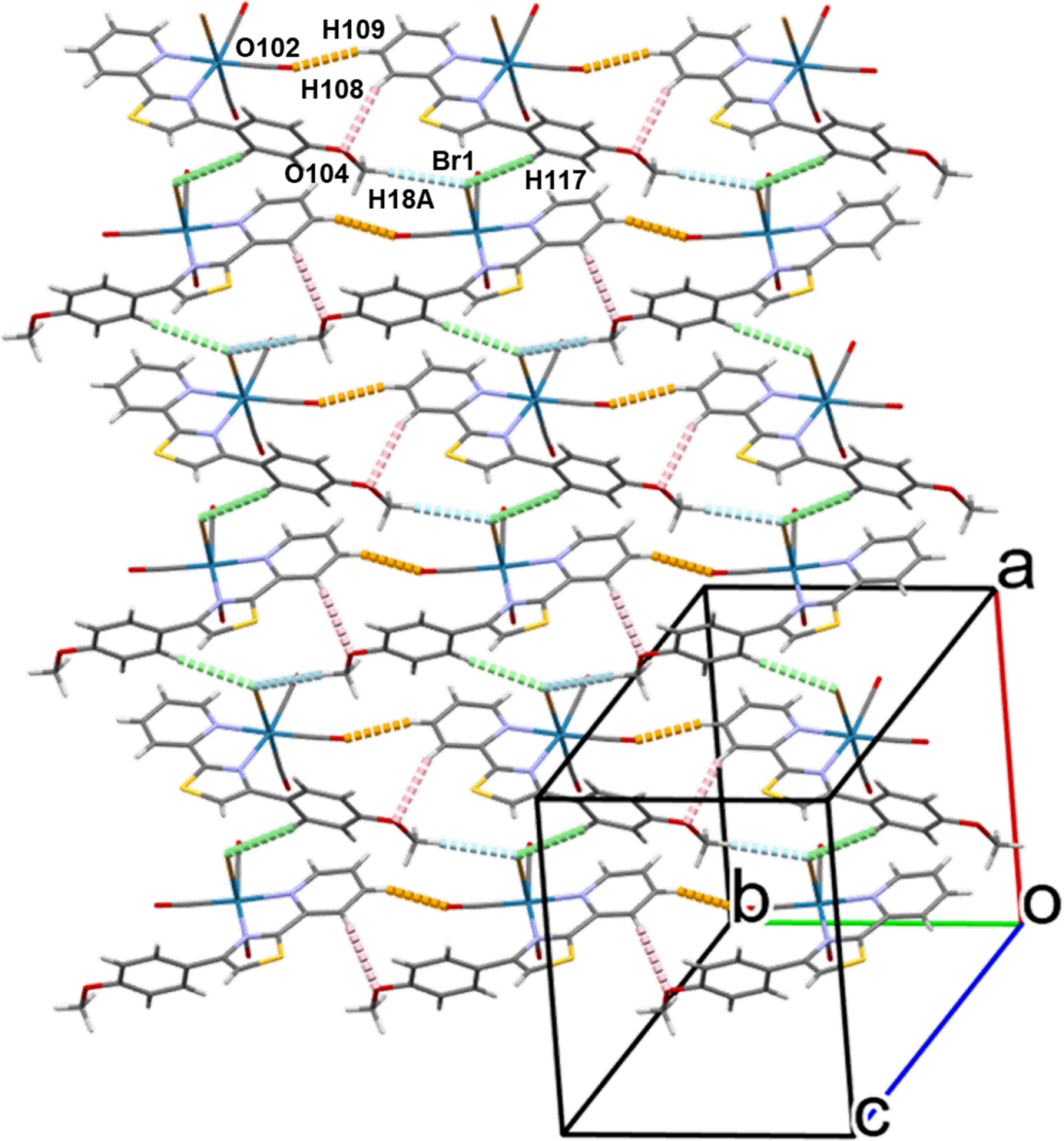
Packing diagram for polymorph β. Hydrogen bonds, C109—H109(py)⋯O102(carbon­yl), C108—H108(py)⋯O104(meth­oxy), C118—H18*A*(methoxy)⋯Br1, and C117—H117(Ph)⋯Br1, are shown as dotted lines in orange, pink, blue, and green, respectively. Color codes: Re (blue); Br (brown); S (yellow); O (red); N (light blue); C (gray); H (light gray).

In polymorph β (Fig. 4 ▸), four types of hydrogen bonds, C108—H108(Py)⋯O104(meth­oxy), C109—H109(Py)⋯O102(carbon­yl), C117—H117(Ph)⋯Br1, and C118—H18*A*(meth­oxy)⋯Br1 (Table 4 ▸), give rise to a di-periodic sheet-like network in the *ab* plane.

## 4. Database survey

A search in Cambridge Structural Database (CSD, Version 5.45, update of November 2023; Groom *et al.*, 2016 ▸) for *fac*-[Re(CO)_3_*X*(*N*^*N*)], where *X* = a halogeno ligand (F, Cl, and Br) and *N*^*N* = chelating ligand or complexing monodentate ligand, yielded 1177 hits, for which *X* = Br gave 441 hits. As for *N*^*N* chelates, compounds coordinated by 2,2′-bi­pyridine and substituted derivatives recorded 78 hits. For the *fac*-[Re(CO)_3_*X*(Py-Th)] complexes (*X* = halogeno ligand; Py-Th = bidentate *N*^*N* ligand containing 2-(pyridin-2-yl)thia­zolyl moiety), 16 crystal structures are available, of which only one structure is found with *X* = Br with the remainder with *X* = Cl. The survey found 62 hits for organic compounds containing the Py-Th backbone (except for transition-metal complexes). Transition-metal complexes chelated by Py-Th ligands include 65, 35, and 20 examples, respectively, for 3*d*, 4*d*, and 5*d*-transition-metal ions.

## 5. Photoluminescence study

**Figure 5:**
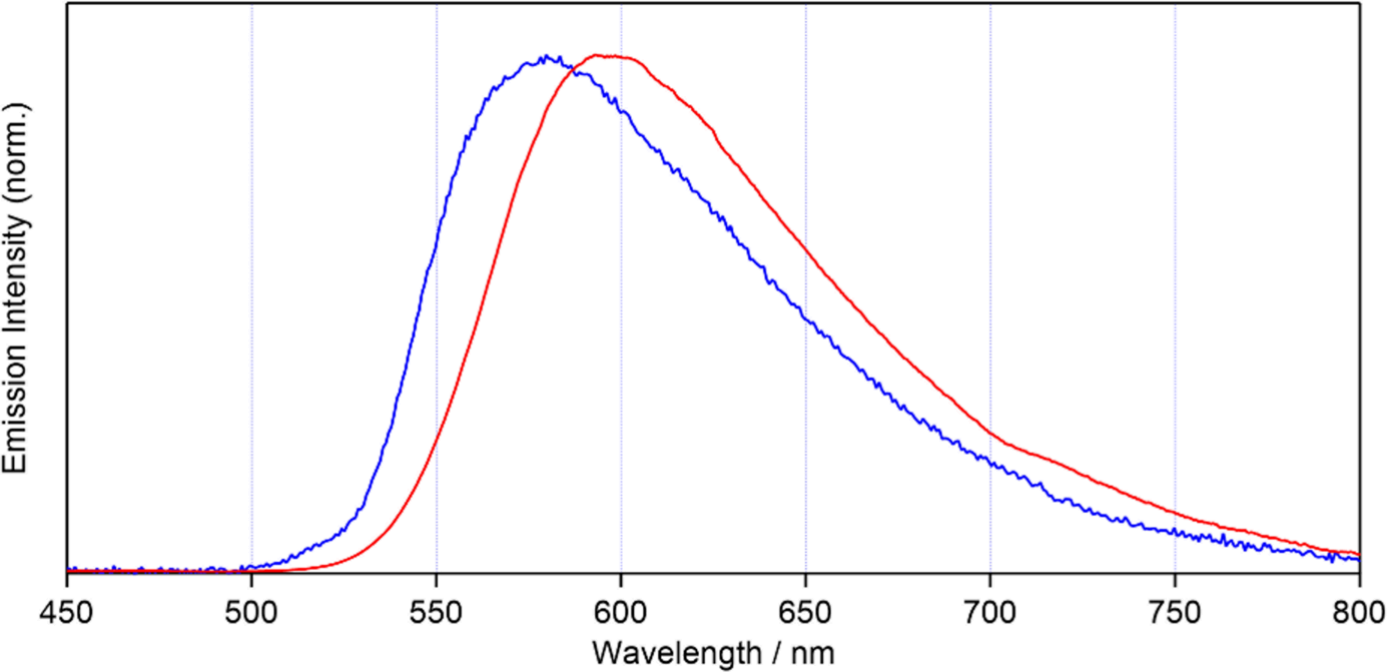
Photoluminescence spectra for polymorphs α (blue line) and β (red line) with *λ*_ex_ = 365 nm.

Upon exposure to UV light at an excitation wavelength (*λ*_ex_) of 365 nm, polymorphs α and β were brightly emissive in yellow and orange, respectively. The solid-state photoluminescence (PL) spectra are depicted in Fig. 5 ▸. The wavelengths of the PL peak maxima (*λ*_PL_) were 580 and 593 nm for polymorphs α and β, respectively, at room temperature, indicating that the crystal-packing variation results in fine-tuning of the PL peak energy. For reference, the PL peak for *fac*-[Re(CO)_3_Br(2,2′-bi­pyridine)] in di­methyl­formamide is observed at *λ*_PL_ = 610 nm (Kutal *et al.*, 1985 ▸).

## 6. Synthesis and crystallization

The ligand ppt-OMe was prepared according to the literature method (Suryawanshi *et al.*, 2018 ▸). Compound (I) was prepared by referring to a previous report (Huff *et al.*, 2016 ▸). An ethano­lic solution (20 ml) of [ReBr(CO)_5_] (166 mg, 0.41 mmol) and ppt-OMe (107 mg, 0.40 mmol) was refluxed for 24 h under an Ar atmosphere. Cooling down the solution to room temperature resulted in precipitation of an orange powdery solid, which was collected by filtration and dried in a vacuum. Yield, 82.5% (based on Re). Recrystallization of the crude solid from CH_2_Cl_2_/*n*-pentane (1/5, *v*/*v*) at room temperature gave one of the two polymorphic forms α (bright yellowish orange, rhomboid-shaped) and β (vivid orange, pillar-shaped) separately in each test tube. ^1^H NMR (CDCl_3_, 600 MHz): δ (ppm) 9.09 (*d*, 1H, py 6-H), 8.09–8.04 (*m*, 2H, py 3,4-H), 7.60 (*td*, 2H, Ph 2,6-H), 7.54–7.52 (*m*, 1H, py 5-H), 7.49 (*s*, 1H, Th), 7.08–7.05 (*m*, 2H, Ph 3,5-H), 3.89 (*s*, 3H, C*H_3_*).

## 7. Refinement

Crystal data, data collection and structure refinement details are summarized in Table 5 ▸. All hydrogen atoms were added at calculated positions and refined using of a riding model with isotropic displacement parameters based on those of the parent atom [C—H = 0.95 Å, *U*_iso_(H) = 1.2*U*_eq_C for CH, C—H = 0.98 Å, *U*_iso_(H) = 1.5*U*_eq_C for CH_3_]. Idealized methyl groups were refined as rotating groups. Inversion twin refinements were applied to polymorph β with a non-centrosymmetric space group in which the absolute structure parameter converged to 0.487 (10).

## Supplementary Material

Crystal structure: contains datablock(s) polymorph-_a, polymorph-_b, publication_text. DOI: 10.1107/S2056989024010727/jp2014sup1.cif

Structure factors: contains datablock(s) polymorph-_a. DOI: 10.1107/S2056989024010727/jp2014polymorph-_asup2.hkl

Structure factors: contains datablock(s) polymorph-_b. DOI: 10.1107/S2056989024010727/jp2014polymorph-_bsup3.hkl

CCDC references: 2400583, 2400582

Additional supporting information:  crystallographic information; 3D view; checkCIF report

## Figures and Tables

**Table 1:** Selected geometric parameters (Å, °) for polymorph α[Chem scheme1]

Re1—C1	1.903 (5)	Re1—Br1	2.6129 (5)
Re1—C2	1.923 (4)	C1—O1	1.145 (5)
Re1—C3	1.950 (5)	C2—O2	1.150 (5)
Re1—N1	2.198 (3)	C3—O3	1.075 (6)
Re1—N2	2.193 (3)		
			
N1—C5—C12—C17	118.0 (4)		

**Table 2:** Selected geometric parameters (Å, °) for polymorph β[Chem scheme1]

Re1—Br1	2.6315 (8)	Re2—C201	1.921 (9)
Re2—Br2	2.6308 (8)	Re2—C202	1.909 (10)
Re1—N101	2.186 (7)	Re2—C203	1.909 (8)
Re1—N102	2.105 (7)	C101—O101	1.153 (11)
Re2—N201	2.185 (7)	C102—O102	1.149 (10)
Re2—N202	2.157 (8)	C103—O103	1.123 (10)
Re1—C101	1.909 (9)	C201—O201	1.137 (11)
Re1—C102	1.919 (9)	C202—O202	1.162 (11)
Re1—C103	1.932 (8)	C203—O203	1.149 (9)
			
N101—C105—C112—C117	61.0 (10)	N201—C205—C212—C217	61.1 (10)

**Table 3:** Hydrogen-bond geometry (Å, °) for polymorph α[Chem scheme1]

*D*—H⋯*A*	*D*—H	H⋯*A*	*D*⋯*A*	*D*—H⋯*A*
C6—H6⋯Br1^i^	0.93	2.80	3.691 (4)	160
C8—H8⋯Br1^ii^	0.93	2.95	3.828 (5)	158
C11—H11⋯O2^iii^	0.93	2.64	3.285 (6)	127
C18—H18*B*⋯O3^iv^	0.96	2.60	3.478 (8)	152

Symmetry codes: (i) 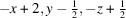; (ii) 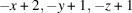; (iii) 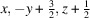; (iv) 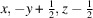.

**Table 4:** Hydrogen-bond geometry (Å, °) for polymorph β[Chem scheme1]

*D*—H⋯*A*	*D*—H	H⋯*A*	*D*⋯*A*	*D*—H⋯*A*
C108—H108⋯O104^i^	0.93	2.66	3.435 (13)	141
C109—H109⋯O102^i^	0.93	2.50	3.397 (14)	161
C208—H208⋯O204^i^	0.93	2.66	3.442 (13)	142
C209—H209⋯O202^i^	0.93	2.43	3.337 (13)	166
C210—H210⋯O101	0.93	2.72	3.110 (12)	106
C214—H214⋯O104^ii^	0.93	2.65	3.365 (12)	135
C117—H117⋯Br1^iii^	0.93	3.03	3.873 (9)	152
C118—H18*A*⋯Br1^iv^	0.96	2.94	3.868 (12)	162
C206—H206⋯Br1^v^	0.93	3.03	3.845 (14)	148
C106—H106⋯Br2^vi^	0.93	3.06	3.762 (14)	133
C218—H18*D*⋯Br2^vii^	0.96	2.97	3.865 (12)	155

Symmetry codes: (i) 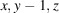; (ii) 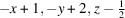; (iii) 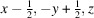; (iv) 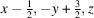; (v) 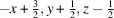; (vi) 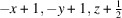; (vii) 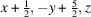.

**Table 5:** Experimental details

	Polymorph α	Polymorph β
Crystal data		
Chemical formula	[ReBr(C_15_H_12_N_2_OS)(CO)_3_]	[ReBr(C_15_H_12_N_2_OS)(CO)_3_]
*M*_r_	618.47	618.47
Crystal system, space group	Monoclinic, *P*2_1_/*c*	Orthorhombic, *Pna*2_1_
Temperature (K)	296	296
*a*, *b*, *c* (Å)	12.7442 (6), 10.6851 (6), 14.4027 (6)	13.2169 (3), 11.2764 (2), 25.8716 (5)
α, β, γ (°)	90, 96.645 (7), 90	90, 90, 90
*V* (Å^3^)	1948.08 (17)	3855.88 (13)
*Z*	4	8
Radiation type	Mo *K*α	Mo *K*α
μ (mm^−1^)	8.42	8.51
Crystal size (mm)	0.48 × 0.38 × 0.26	0.68 × 0.2 × 0.1

Data collection		
Diffractometer	Rigaku R-Axis Rapid	Rigaku R-Axis Rapid
Absorption correction	Multi-scan (*ABSCOR*; Higashi, 1995 ▸)	Multi-scan *ABSCOR* (Higashi, 1995 ▸)
*T*_min_, *T*_max_	0.373, 1	0.318, 1
No. of measured, independent and observed [*I* > 2σ(*I*)] reflections	22185, 5657, 4722	69569, 11206, 9179
*R*_int_	0.035	0.055
(sin θ/λ)_max_ (Å^−1^)	0.703	0.703

Refinement		
*R*[*F*^2^ > 2σ(*F*^2^)], *wR*(*F*^2^), *S*	0.034, 0.079, 1.13	0.033, 0.065, 1.02
No. of reflections	5657	11206
No. of parameters	244	488
No. of restraints	0	1
H-atom treatment	H-atom parameters constrained	H-atom parameters constrained
Δρ_max_, Δρ_min_ (e Å^−3^)	2.32, −0.60	2.38, −0.50
Absolute structure	–	Refined as an inversion twin.
Absolute structure parameter	–	0.487 (10)

Computer programs: *RAPID-AUTO* (Rigaku, 2006 ▸), *SORTAV* (Blessing, 1995 ▸), *SHELXT2018/2* (Sheldrick, 2015*a* ▸), *SHELXL2018/3* (Sheldrick, 2015*b* ▸), *PLATON* (Spek, 2020 ▸) and *WinGX* publication routines (Farrugia, 2012 ▸).

## supplementary crystallographic information

### fac-Bromidotricarbonyl[4-(4-methoxyphenyl)-2-(pyridin-2-yl)thiazole-κ2N,N']rhenium(I) (polymorph-_a) Crystal data

**Table:**

[ReBr(C_15_H_12_N_2_OS)(CO)_3_]	*F*(000) = 1168
*M**_r_* = 618.47	*D*_x_ = 2.109 Mg m^−^^3^
Monoclinic, *P*2_1_/*c*	Mo *K*α radiation, λ = 0.71073 Å
*a* = 12.7442 (6) Å	Cell parameters from 18153 reflections
*b* = 10.6851 (6) Å	θ = 2.3–30.0°
*c* = 14.4027 (6) Å	µ = 8.42 mm^−^^1^
β = 96.645 (7)°	*T* = 296 K
*V* = 1948.08 (17) Å^3^	Rhomboid, bright yellowish orange
*Z* = 4	0.48 × 0.38 × 0.26 mm

### fac-Bromidotricarbonyl[4-(4-methoxyphenyl)-2-(pyridin-2-yl)thiazole-κ2N,N']rhenium(I) (polymorph-_a) Data collection

**Table:**

Rigaku R-Axis Rapid diffractometer	5657 independent reflections
Radiation source: sealed x-ray tube	4722 reflections with *I* > 2σ(*I*)
Graphite monochromator	*R*_int_ = 0.035
Detector resolution: 10 pixels mm^-1^	θ_max_ = 30.0°, θ_min_ = 2.4°
ω oscillation scans	*h* = −16→17
Absorption correction: multi-scan (ABSCOR; Higashi, 1995)	*k* = −15→15
*T*_min_ = 0.373, *T*_max_ = 1	*l* = −20→20
22185 measured reflections	

### fac-Bromidotricarbonyl[4-(4-methoxyphenyl)-2-(pyridin-2-yl)thiazole-κ2N,N']rhenium(I) (polymorph-_a) Refinement

**Table:**

Refinement on *F*^2^	Primary atom site location: dual
Least-squares matrix: full	Secondary atom site location: dual
*R*[*F*^2^ > 2σ(*F*^2^)] = 0.034	Hydrogen site location: inferred from neighbouring sites
*wR*(*F*^2^) = 0.079	H-atom parameters constrained
*S* = 1.13	*w* = 1/[σ^2^(*F*_o_^2^) + (0.0414*P*)^2^ + 0.8663*P*] where *P* = (*F*_o_^2^ + 2*F*_c_^2^)/3
5657 reflections	(Δ/σ)_max_ = 0.002
244 parameters	Δρ_max_ = 2.32 e Å^−^^3^
0 restraints	Δρ_min_ = −0.60 e Å^−^^3^
0 constraints	

### fac-Bromidotricarbonyl[4-(4-methoxyphenyl)-2-(pyridin-2-yl)thiazole-κ2N,N']rhenium(I) (polymorph-_a) Special details

**Table:**

Geometry. All esds (except the esd in the dihedral angle between two l.s. planes) are estimated using the full covariance matrix. The cell esds are taken into account individually in the estimation of esds in distances, angles and torsion angles; correlations between esds in cell parameters are only used when they are defined by crystal symmetry. An approximate (isotropic) treatment of cell esds is used for estimating esds involving l.s. planes.

### fac-Bromidotricarbonyl[4-(4-methoxyphenyl)-2-(pyridin-2-yl)thiazole-κ2N,N']rhenium(I) (polymorph-_a) Fractional atomic coordinates and isotropic or equivalent isotropic displacement parameters (Å^2^)

**Table:**

	*x*	*y*	*z*	*U*_iso_*/*U*_eq_	
Re1	0.69332 (2)	0.57379 (2)	0.34624 (2)	0.03362 (6)	
Br1	0.85382 (3)	0.72741 (4)	0.35771 (3)	0.04761 (11)	
C1	0.5981 (4)	0.7040 (5)	0.3700 (3)	0.0522 (11)	
C2	0.6607 (3)	0.6114 (4)	0.2155 (3)	0.0410 (9)	
C3	0.5766 (4)	0.4552 (5)	0.3419 (3)	0.0487 (10)	
O4	0.7290 (3)	0.4398 (4)	−0.1125 (2)	0.0692 (11)	
O1	0.5395 (4)	0.7803 (4)	0.3856 (3)	0.0912 (14)	
O2	0.6396 (3)	0.6374 (3)	0.1381 (2)	0.0606 (9)	
O3	0.5101 (3)	0.3926 (4)	0.3360 (3)	0.0765 (11)	
S1	0.97094 (8)	0.30377 (10)	0.42134 (7)	0.0430 (2)	
N1	0.8157 (3)	0.4295 (3)	0.3425 (2)	0.0336 (6)	
N2	0.7479 (3)	0.5328 (3)	0.4931 (2)	0.0373 (7)	
C4	0.8643 (3)	0.3978 (3)	0.4256 (3)	0.0347 (8)	
C5	0.8644 (3)	0.3775 (4)	0.2704 (3)	0.0350 (7)	
C6	0.9495 (3)	0.3080 (4)	0.3017 (3)	0.0430 (9)	
H6	0.991785	0.266982	0.262858	0.052*	
C7	0.8276 (3)	0.4487 (3)	0.5101 (3)	0.0338 (8)	
C8	0.8708 (4)	0.4192 (4)	0.5996 (3)	0.0445 (9)	
H8	0.925785	0.361826	0.609453	0.053*	
C9	0.8314 (4)	0.4758 (5)	0.6745 (3)	0.0502 (10)	
H9	0.859435	0.456710	0.735313	0.060*	
C10	0.7512 (4)	0.5600 (4)	0.6582 (3)	0.0496 (11)	
H10	0.723744	0.599092	0.707814	0.060*	
C11	0.7108 (4)	0.5868 (4)	0.5663 (3)	0.0480 (10)	
H11	0.656012	0.644385	0.555517	0.058*	
C12	0.8245 (3)	0.3970 (4)	0.1715 (3)	0.0354 (8)	
C13	0.7276 (3)	0.3513 (4)	0.1340 (3)	0.0453 (10)	
H13	0.684776	0.311444	0.172987	0.054*	
C14	0.6921 (3)	0.3633 (4)	0.0395 (3)	0.0473 (10)	
H14	0.626769	0.330859	0.015642	0.057*	
C15	0.7547 (4)	0.4239 (4)	−0.0189 (3)	0.0464 (10)	
C16	0.8511 (4)	0.4715 (6)	0.0181 (3)	0.0576 (12)	
H16	0.892889	0.513764	−0.020479	0.069*	
C17	0.8861 (4)	0.4574 (5)	0.1110 (3)	0.0490 (10)	
H17	0.952105	0.488540	0.134264	0.059*	
C18	0.6257 (6)	0.4026 (7)	−0.1508 (4)	0.084 (2)	
H18A	0.616800	0.418214	−0.216846	0.125*	
H18B	0.616401	0.315003	−0.139528	0.125*	
H18C	0.574212	0.449674	−0.121700	0.125*	

### fac-Bromidotricarbonyl[4-(4-methoxyphenyl)-2-(pyridin-2-yl)thiazole-κ2N,N']rhenium(I) (polymorph-_a) Atomic displacement parameters (Å^2^)

**Table:**

	*U*^11^	*U*^22^	*U*^33^	*U*^12^	*U*^13^	*U*^23^
Re1	0.02619 (8)	0.04068 (9)	0.03484 (9)	0.00630 (6)	0.00720 (6)	0.00405 (6)
Br1	0.0396 (2)	0.0519 (2)	0.0510 (2)	−0.00480 (18)	0.00401 (17)	0.00717 (18)
C1	0.041 (2)	0.063 (3)	0.053 (3)	0.014 (2)	0.0099 (19)	0.007 (2)
C2	0.033 (2)	0.042 (2)	0.048 (2)	0.0057 (16)	0.0046 (16)	0.0072 (17)
C3	0.029 (2)	0.055 (3)	0.059 (3)	0.0102 (19)	−0.0049 (18)	−0.010 (2)
O4	0.060 (2)	0.110 (3)	0.0371 (17)	−0.019 (2)	0.0011 (16)	0.0031 (17)
O1	0.082 (3)	0.091 (3)	0.105 (3)	0.056 (3)	0.031 (2)	0.010 (2)
O2	0.069 (2)	0.066 (2)	0.0453 (18)	0.0122 (18)	−0.0008 (15)	0.0115 (15)
O3	0.054 (2)	0.077 (3)	0.101 (3)	−0.008 (2)	0.017 (2)	0.014 (2)
S1	0.0368 (5)	0.0493 (6)	0.0428 (5)	0.0140 (4)	0.0038 (4)	0.0015 (4)
N1	0.0330 (17)	0.0363 (16)	0.0323 (16)	0.0020 (12)	0.0079 (12)	0.0008 (11)
N2	0.0331 (17)	0.0436 (17)	0.0371 (16)	0.0056 (14)	0.0119 (13)	0.0028 (13)
C4	0.0321 (19)	0.0341 (18)	0.039 (2)	0.0040 (14)	0.0075 (15)	0.0040 (14)
C5	0.0325 (19)	0.0390 (19)	0.0352 (18)	0.0005 (15)	0.0106 (14)	−0.0033 (14)
C6	0.038 (2)	0.055 (2)	0.038 (2)	0.0123 (18)	0.0104 (16)	−0.0042 (17)
C7	0.0309 (18)	0.0369 (19)	0.0347 (18)	0.0011 (14)	0.0087 (14)	0.0042 (13)
C8	0.044 (2)	0.050 (2)	0.040 (2)	0.0031 (18)	0.0054 (17)	0.0067 (16)
C9	0.050 (3)	0.069 (3)	0.032 (2)	−0.002 (2)	0.0051 (17)	0.0037 (19)
C10	0.058 (3)	0.058 (3)	0.036 (2)	0.001 (2)	0.0163 (19)	−0.0051 (17)
C11	0.048 (3)	0.056 (3)	0.043 (2)	0.0115 (19)	0.0137 (19)	−0.0020 (18)
C12	0.0289 (18)	0.042 (2)	0.0371 (19)	0.0030 (14)	0.0099 (14)	−0.0045 (14)
C13	0.037 (2)	0.059 (3)	0.041 (2)	−0.0078 (19)	0.0103 (17)	0.0043 (18)
C14	0.039 (2)	0.057 (3)	0.045 (2)	−0.0103 (19)	0.0010 (17)	0.0004 (19)
C15	0.042 (2)	0.061 (3)	0.036 (2)	−0.0012 (19)	0.0045 (17)	−0.0018 (17)
C16	0.045 (3)	0.086 (4)	0.042 (2)	−0.018 (2)	0.0109 (19)	0.006 (2)
C17	0.037 (2)	0.068 (3)	0.043 (2)	−0.012 (2)	0.0084 (18)	0.0003 (19)
C18	0.083 (5)	0.109 (5)	0.053 (3)	−0.029 (4)	−0.021 (3)	0.013 (3)

### fac-Bromidotricarbonyl[4-(4-methoxyphenyl)-2-(pyridin-2-yl)thiazole-κ2N,N']rhenium(I) (polymorph-_a) Geometric parameters (Å, º)

**Table:**

Re1—C1	1.903 (5)	C7—C8	1.379 (5)
Re1—C2	1.923 (4)	C8—C9	1.380 (6)
Re1—C3	1.950 (5)	C8—H8	0.9300
Re1—N2	2.193 (3)	C9—C10	1.361 (7)
Re1—N1	2.198 (3)	C9—H9	0.9300
Re1—Br1	2.6129 (5)	C10—C11	1.394 (6)
C1—O1	1.145 (5)	C10—H10	0.9300
C2—O2	1.150 (5)	C11—H11	0.9300
C3—O3	1.075 (6)	C12—C13	1.379 (5)
O4—C15	1.361 (5)	C12—C17	1.397 (5)
O4—C18	1.424 (7)	C13—C14	1.389 (6)
S1—C4	1.697 (4)	C13—H13	0.9300
S1—C6	1.713 (4)	C14—C15	1.385 (6)
N1—C4	1.327 (5)	C14—H14	0.9300
N1—C5	1.387 (5)	C15—C16	1.379 (6)
N2—C11	1.336 (5)	C16—C17	1.368 (6)
N2—C7	1.356 (5)	C16—H16	0.9300
C4—C7	1.458 (5)	C17—H17	0.9300
C5—C6	1.350 (5)	C18—H18A	0.9600
C5—C12	1.470 (5)	C18—H18B	0.9600
C6—H6	0.9300	C18—H18C	0.9600
			
C1—Re1—C2	87.31 (18)	C8—C7—C4	124.3 (4)
C1—Re1—C3	88.9 (2)	C7—C8—C9	119.3 (4)
C2—Re1—C3	91.56 (19)	C7—C8—H8	120.4
C1—Re1—N2	96.34 (16)	C9—C8—H8	120.4
C2—Re1—N2	174.02 (14)	C10—C9—C8	119.3 (4)
C3—Re1—N2	93.25 (17)	C10—C9—H9	120.4
C1—Re1—N1	170.47 (17)	C8—C9—H9	120.4
C2—Re1—N1	101.33 (15)	C9—C10—C11	119.1 (4)
C3—Re1—N1	94.84 (16)	C9—C10—H10	120.4
N2—Re1—N1	74.72 (12)	C11—C10—H10	120.4
C1—Re1—Br1	92.31 (15)	N2—C11—C10	122.3 (4)
C2—Re1—Br1	90.54 (13)	N2—C11—H11	118.8
C3—Re1—Br1	177.63 (14)	C10—C11—H11	118.8
N2—Re1—Br1	84.59 (9)	C13—C12—C17	117.5 (4)
N1—Re1—Br1	83.67 (8)	C13—C12—C5	121.6 (3)
O1—C1—Re1	178.3 (5)	C17—C12—C5	120.9 (4)
O2—C2—Re1	177.8 (4)	C12—C13—C14	121.8 (4)
O3—C3—Re1	176.7 (5)	C12—C13—H13	119.1
C15—O4—C18	117.0 (4)	C14—C13—H13	119.1
C4—S1—C6	89.20 (19)	C15—C14—C13	119.5 (4)
C4—N1—C5	111.8 (3)	C15—C14—H14	120.2
C4—N1—Re1	114.6 (2)	C13—C14—H14	120.2
C5—N1—Re1	132.8 (3)	O4—C15—C16	116.0 (4)
C11—N2—C7	118.1 (3)	O4—C15—C14	124.8 (4)
C11—N2—Re1	124.9 (3)	C16—C15—C14	119.2 (4)
C7—N2—Re1	117.0 (2)	C17—C16—C15	120.8 (4)
N1—C4—C7	119.7 (3)	C17—C16—H16	119.6
N1—C4—S1	114.3 (3)	C15—C16—H16	119.6
C7—C4—S1	125.9 (3)	C16—C17—C12	121.2 (4)
C6—C5—N1	112.5 (3)	C16—C17—H17	119.4
C6—C5—C12	125.2 (3)	C12—C17—H17	119.4
N1—C5—C12	122.3 (3)	O4—C18—H18A	109.5
C5—C6—S1	112.3 (3)	O4—C18—H18B	109.5
C5—C6—H6	123.9	H18A—C18—H18B	109.5
S1—C6—H6	123.9	O4—C18—H18C	109.5
N2—C7—C8	122.0 (4)	H18A—C18—H18C	109.5
N2—C7—C4	113.7 (3)	H18B—C18—H18C	109.5
			
C5—N1—C4—C7	176.9 (3)	C4—C7—C8—C9	178.2 (4)
Re1—N1—C4—C7	6.1 (4)	C7—C8—C9—C10	−0.3 (7)
C5—N1—C4—S1	0.0 (4)	C8—C9—C10—C11	0.0 (7)
Re1—N1—C4—S1	−170.94 (17)	C7—N2—C11—C10	0.2 (7)
C6—S1—C4—N1	0.3 (3)	Re1—N2—C11—C10	−177.7 (3)
C6—S1—C4—C7	−176.5 (4)	C9—C10—C11—N2	0.0 (7)
C4—N1—C5—C6	−0.4 (5)	C6—C5—C12—C13	114.1 (5)
Re1—N1—C5—C6	168.3 (3)	N1—C5—C12—C13	−65.2 (5)
C4—N1—C5—C12	179.0 (4)	C6—C5—C12—C17	−62.7 (6)
Re1—N1—C5—C12	−12.3 (6)	N1—C5—C12—C17	118.0 (4)
N1—C5—C6—S1	0.6 (5)	C17—C12—C13—C14	0.6 (6)
C12—C5—C6—S1	−178.7 (3)	C5—C12—C13—C14	−176.3 (4)
C4—S1—C6—C5	−0.5 (3)	C12—C13—C14—C15	−0.8 (7)
C11—N2—C7—C8	−0.5 (6)	C18—O4—C15—C16	−173.9 (5)
Re1—N2—C7—C8	177.6 (3)	C18—O4—C15—C14	6.8 (8)
C11—N2—C7—C4	−178.4 (4)	C13—C14—C15—O4	179.1 (4)
Re1—N2—C7—C4	−0.3 (4)	C13—C14—C15—C16	−0.2 (7)
N1—C4—C7—N2	−3.9 (5)	O4—C15—C16—C17	−178.0 (5)
S1—C4—C7—N2	172.7 (3)	C14—C15—C16—C17	1.3 (8)
N1—C4—C7—C8	178.2 (4)	C15—C16—C17—C12	−1.5 (8)
S1—C4—C7—C8	−5.1 (6)	C13—C12—C17—C16	0.5 (7)
N2—C7—C8—C9	0.6 (6)	C5—C12—C17—C16	177.4 (5)

### fac-Bromidotricarbonyl[4-(4-methoxyphenyl)-2-(pyridin-2-yl)thiazole-κ2N,N']rhenium(I) (polymorph-_a) Hydrogen-bond geometry (Å, º)

**Table:**

*D*—H···*A*	*D*—H	H···*A*	*D*···*A*	*D*—H···*A*
C6—H6···Br1^i^	0.93	2.80	3.691 (4)	160
C8—H8···Br1^ii^	0.93	2.95	3.828 (5)	158
C11—H11···O2^iii^	0.93	2.64	3.285 (6)	127
C18—H18*B*···O3^iv^	0.96	2.60	3.478 (8)	152

Symmetry codes: (i) −*x*+2, *y*−1/2, −*z*+1/2; (ii) −*x*+2, −*y*+1, −*z*+1; (iii) *x*, −*y*+3/2, *z*+1/2; (iv) *x*, −*y*+1/2, *z*−1/2.

### fac-Bromidotricarbonyl[4-(4-methoxyphenyl)-2-(pyridin-2-yl)thiazole-κ2N,N']rhenium(I) (polymorph-_b) Crystal data

**Table:**

[ReBr(C_15_H_12_N_2_OS)(CO)_3_]	*D*_x_ = 2.131 Mg m^−^^3^
*M**_r_* = 618.47	Mo *K*α radiation, λ = 0.71073 Å
Orthorhombic, *P**n**a*2_1_	Cell parameters from 52848 reflections
*a* = 13.2169 (3) Å	θ = 1.7–30.0°
*b* = 11.2764 (2) Å	µ = 8.51 mm^−^^1^
*c* = 25.8716 (5) Å	*T* = 296 K
*V* = 3855.88 (13) Å^3^	Pillar, vivid orange
*Z* = 8	0.68 × 0.2 × 0.1 mm
*F*(000) = 2336	

### fac-Bromidotricarbonyl[4-(4-methoxyphenyl)-2-(pyridin-2-yl)thiazole-κ2N,N']rhenium(I) (polymorph-_b) Data collection

**Table:**

Rigaku R-Axis Rapid diffractometer	11206 independent reflections
Radiation source: sealed x-ray tube	9179 reflections with *I* > 2σ(*I*)
Graphite monochromator	*R*_int_ = 0.055
Detector resolution: 10 pixels mm^-1^	θ_max_ = 30.0°, θ_min_ = 2.4°
ω oscillation scans	*h* = −18→18
Absorption correction: multi-scan ABSCOR (Higashi, 1995)	*k* = −15→15
*T*_min_ = 0.318, *T*_max_ = 1	*l* = −36→36
69569 measured reflections	

### fac-Bromidotricarbonyl[4-(4-methoxyphenyl)-2-(pyridin-2-yl)thiazole-κ2N,N']rhenium(I) (polymorph-_b) Refinement

**Table:**

Refinement on *F*^2^	Hydrogen site location: inferred from neighbouring sites
Least-squares matrix: full	H-atom parameters constrained
*R*[*F*^2^ > 2σ(*F*^2^)] = 0.033	*w* = 1/[σ^2^(*F*_o_^2^) + (0.0351*P*)^2^] where *P* = (*F*_o_^2^ + 2*F*_c_^2^)/3
*wR*(*F*^2^) = 0.065	(Δ/σ)_max_ = 0.001
*S* = 1.02	Δρ_max_ = 2.38 e Å^−^^3^
11206 reflections	Δρ_min_ = −0.49 e Å^−^^3^
488 parameters	Absolute structure: Refined as an inversion twin.
1 restraint	Absolute structure parameter: 0.487 (10)
0 constraints	

### fac-Bromidotricarbonyl[4-(4-methoxyphenyl)-2-(pyridin-2-yl)thiazole-κ2N,N']rhenium(I) (polymorph-_b) Special details

**Table:**

Geometry. All esds (except the esd in the dihedral angle between two l.s. planes) are estimated using the full covariance matrix. The cell esds are taken into account individually in the estimation of esds in distances, angles and torsion angles; correlations between esds in cell parameters are only used when they are defined by crystal symmetry. An approximate (isotropic) treatment of cell esds is used for estimating esds involving l.s. planes.
Refinement. Refined as a 2-component inversion twin

### fac-Bromidotricarbonyl[4-(4-methoxyphenyl)-2-(pyridin-2-yl)thiazole-κ2N,N']rhenium(I) (polymorph-_b) Fractional atomic coordinates and isotropic or equivalent isotropic displacement parameters (Å^2^)

**Table:**

	*x*	*y*	*z*	*U*_iso_*/*U*_eq_	
Re1	0.42111 (2)	0.32777 (3)	0.57274 (2)	0.03102 (7)	
Re2	0.81648 (2)	0.82562 (3)	0.42520 (2)	0.03003 (7)	
Br1	0.59268 (6)	0.31952 (8)	0.62422 (4)	0.0463 (2)	
Br2	0.64622 (6)	0.81674 (7)	0.37267 (4)	0.04512 (19)	
C101	0.4884 (6)	0.3571 (9)	0.5087 (4)	0.044 (2)	
C102	0.4162 (5)	0.4955 (8)	0.5850 (3)	0.034 (2)	
C103	0.2921 (6)	0.3320 (7)	0.5377 (3)	0.0377 (17)	
C201	0.7481 (6)	0.8531 (9)	0.4896 (4)	0.044 (2)	
C202	0.8197 (5)	0.9933 (9)	0.4153 (4)	0.041 (2)	
C203	0.9428 (6)	0.8350 (7)	0.4608 (3)	0.0367 (17)	
O101	0.5286 (5)	0.3730 (8)	0.4698 (3)	0.073 (2)	
O102	0.4168 (5)	0.5965 (6)	0.5907 (3)	0.054 (2)	
O103	0.2190 (5)	0.3408 (6)	0.5159 (3)	0.0567 (17)	
O104	0.2265 (5)	0.8047 (6)	0.6793 (3)	0.0572 (18)	
O201	0.7076 (5)	0.8627 (8)	0.5282 (3)	0.072 (2)	
O204	1.0177 (5)	1.3088 (6)	0.3140 (3)	0.0603 (19)	
O202	0.8169 (5)	1.0953 (6)	0.4091 (3)	0.056 (2)	
O203	1.0185 (4)	0.8387 (6)	0.4825 (3)	0.0518 (16)	
S1	0.34498 (19)	0.1014 (3)	0.71414 (11)	0.0583 (7)	
S2	0.90592 (19)	0.6057 (3)	0.28377 (11)	0.0551 (6)	
N101	0.3575 (4)	0.2621 (6)	0.6453 (3)	0.0350 (16)	
N102	0.4408 (4)	0.1427 (6)	0.5690 (3)	0.0319 (15)	
N201	0.8824 (4)	0.7637 (6)	0.3526 (3)	0.0317 (14)	
N202	0.8055 (5)	0.6348 (7)	0.4266 (5)	0.0485 (18)	
C104	0.3707 (6)	0.1469 (8)	0.6523 (3)	0.0414 (19)	
C105	0.3240 (6)	0.3178 (8)	0.6900 (4)	0.040 (2)	
C106	0.3157 (6)	0.2441 (10)	0.7310 (5)	0.053 (3)	
H106	0.296607	0.267741	0.764048	0.063*	
C107	0.4075 (6)	0.0731 (8)	0.6103 (4)	0.047 (2)	
C108	0.4128 (7)	−0.0497 (10)	0.6119 (5)	0.058 (3)	
H108	0.390653	−0.091276	0.640778	0.070*	
C109	0.4514 (7)	−0.1090 (9)	0.5697 (7)	0.067 (3)	
H109	0.454297	−0.191362	0.569745	0.080*	
C110	0.4853 (7)	−0.0472 (9)	0.5278 (5)	0.063 (3)	
H110	0.514055	−0.085687	0.499587	0.076*	
C111	0.4753 (6)	0.0751 (9)	0.5289 (4)	0.054 (2)	
H111	0.494083	0.115576	0.499105	0.064*	
C112	0.2953 (6)	0.4467 (8)	0.6879 (3)	0.0381 (18)	
C113	0.3431 (7)	0.5308 (9)	0.7179 (4)	0.054 (2)	
H113	0.393804	0.507262	0.740541	0.065*	
C114	0.3174 (8)	0.6485 (11)	0.7150 (5)	0.059 (3)	
H114	0.350450	0.703600	0.735717	0.071*	
C115	0.2424 (7)	0.6855 (8)	0.6812 (4)	0.042 (2)	
C116	0.1912 (6)	0.6032 (8)	0.6521 (4)	0.0383 (19)	
H116	0.139678	0.626817	0.629940	0.046*	
C117	0.2177 (6)	0.4823 (8)	0.6562 (3)	0.0439 (19)	
H117	0.182156	0.426051	0.637186	0.053*	
C118	0.1471 (10)	0.8468 (11)	0.6449 (5)	0.077 (4)	
H18A	0.143045	0.931663	0.646860	0.116*	
H18B	0.162437	0.823563	0.610069	0.116*	
H18C	0.083544	0.812885	0.655076	0.116*	
C204	0.8733 (6)	0.6499 (7)	0.3446 (3)	0.0408 (19)	
C205	0.9186 (5)	0.8245 (8)	0.3099 (3)	0.037 (2)	
C206	0.9333 (6)	0.7506 (8)	0.2692 (5)	0.049 (3)	
H206	0.955794	0.775727	0.236910	0.059*	
C207	0.8349 (5)	0.5739 (8)	0.3859 (4)	0.043 (2)	
C208	0.8297 (7)	0.4493 (8)	0.3828 (5)	0.061 (3)	
H208	0.852637	0.409643	0.353522	0.073*	
C209	0.7902 (8)	0.3876 (8)	0.4239 (7)	0.068 (3)	
H209	0.787306	0.305171	0.423054	0.081*	
C210	0.7546 (7)	0.4493 (9)	0.4667 (5)	0.065 (3)	
H210	0.726145	0.409378	0.494541	0.078*	
C211	0.7625 (6)	0.5696 (8)	0.4668 (4)	0.050 (2)	
H211	0.737728	0.610608	0.495283	0.060*	
C212	0.9437 (5)	0.9508 (8)	0.3119 (3)	0.0349 (17)	
C213	0.8936 (6)	1.0346 (9)	0.2805 (4)	0.051 (2)	
H213	0.842625	1.010803	0.257986	0.061*	
C214	0.9213 (7)	1.1524 (9)	0.2836 (4)	0.050 (2)	
H214	0.887457	1.207597	0.263199	0.060*	
C215	0.9973 (6)	1.1906 (8)	0.3158 (4)	0.044 (2)	
C216	1.0462 (6)	1.1085 (10)	0.3460 (3)	0.047 (2)	
H216	1.098881	1.132535	0.367428	0.056*	
C217	1.0182 (5)	0.9917 (8)	0.3449 (3)	0.0392 (17)	
H217	1.050094	0.938399	0.367014	0.047*	
C218	1.0987 (10)	1.3514 (11)	0.3443 (6)	0.085 (4)	
H18D	1.105112	1.435523	0.339621	0.128*	
H18E	1.085756	1.334564	0.380088	0.128*	
H18F	1.160269	1.313154	0.333876	0.128*	

### fac-Bromidotricarbonyl[4-(4-methoxyphenyl)-2-(pyridin-2-yl)thiazole-κ2N,N']rhenium(I) (polymorph-_b) Atomic displacement parameters (Å^2^)

**Table:**

	*U*^11^	*U*^22^	*U*^33^	*U*^12^	*U*^13^	*U*^23^
Re1	0.03245 (13)	0.02514 (14)	0.03548 (15)	0.00021 (11)	−0.00028 (13)	0.0014 (2)
Re2	0.03197 (13)	0.02439 (14)	0.03373 (14)	−0.00033 (10)	0.00011 (13)	−0.0005 (2)
Br1	0.0383 (4)	0.0416 (5)	0.0590 (5)	0.0002 (3)	−0.0111 (4)	−0.0010 (4)
Br2	0.0371 (4)	0.0420 (5)	0.0563 (5)	−0.0006 (3)	−0.0104 (3)	0.0012 (4)
C101	0.044 (4)	0.044 (5)	0.045 (5)	0.005 (4)	−0.002 (4)	0.003 (4)
C102	0.031 (3)	0.034 (4)	0.038 (6)	−0.005 (3)	−0.002 (3)	0.010 (3)
C103	0.039 (4)	0.031 (4)	0.043 (4)	0.002 (4)	0.004 (4)	0.000 (3)
C201	0.038 (4)	0.052 (5)	0.042 (5)	0.000 (4)	−0.002 (4)	−0.003 (4)
C202	0.038 (4)	0.044 (5)	0.042 (7)	0.001 (3)	−0.003 (3)	−0.006 (4)
C203	0.043 (4)	0.033 (4)	0.034 (4)	−0.002 (3)	−0.004 (3)	−0.003 (3)
O101	0.066 (4)	0.106 (6)	0.047 (4)	0.007 (4)	0.019 (3)	0.016 (4)
O102	0.069 (4)	0.019 (3)	0.072 (6)	−0.005 (3)	0.004 (3)	−0.002 (3)
O103	0.044 (3)	0.065 (5)	0.061 (4)	0.001 (3)	−0.011 (3)	0.007 (3)
O104	0.072 (4)	0.039 (4)	0.061 (4)	0.014 (3)	−0.004 (3)	−0.006 (3)
O201	0.066 (4)	0.102 (6)	0.049 (4)	−0.008 (4)	0.016 (3)	−0.006 (4)
O204	0.061 (4)	0.045 (4)	0.075 (5)	−0.006 (3)	−0.008 (4)	−0.005 (4)
O202	0.074 (4)	0.032 (4)	0.062 (5)	0.003 (3)	0.005 (3)	−0.005 (3)
O203	0.043 (3)	0.057 (4)	0.055 (4)	−0.002 (3)	−0.012 (3)	−0.002 (3)
S1	0.0505 (12)	0.0546 (15)	0.0699 (17)	−0.0037 (12)	0.0001 (12)	0.0348 (14)
S2	0.0574 (13)	0.0508 (15)	0.0571 (14)	0.0089 (12)	−0.0030 (12)	−0.0264 (12)
N101	0.031 (3)	0.028 (4)	0.046 (5)	0.001 (3)	0.005 (3)	0.006 (3)
N102	0.019 (2)	0.047 (4)	0.030 (3)	0.017 (2)	0.006 (3)	0.009 (4)
N201	0.030 (3)	0.031 (4)	0.034 (4)	0.001 (3)	−0.001 (3)	−0.009 (3)
N202	0.047 (4)	0.038 (4)	0.060 (5)	0.022 (3)	−0.017 (4)	0.007 (5)
C104	0.034 (4)	0.034 (4)	0.055 (5)	−0.003 (3)	−0.002 (4)	0.014 (4)
C105	0.036 (4)	0.044 (6)	0.039 (5)	0.002 (3)	−0.002 (3)	0.011 (4)
C106	0.044 (5)	0.074 (9)	0.041 (8)	0.006 (4)	0.006 (4)	0.023 (5)
C107	0.038 (4)	0.027 (4)	0.077 (7)	−0.006 (4)	−0.012 (4)	0.007 (4)
C108	0.051 (5)	0.040 (6)	0.085 (8)	−0.004 (4)	−0.015 (5)	0.013 (5)
C109	0.064 (6)	0.029 (5)	0.109 (10)	0.002 (4)	−0.016 (8)	−0.006 (7)
C110	0.058 (5)	0.043 (6)	0.089 (8)	0.008 (4)	−0.017 (5)	−0.020 (6)
C111	0.042 (4)	0.046 (5)	0.073 (6)	−0.003 (4)	−0.014 (4)	−0.006 (5)
C112	0.039 (4)	0.044 (5)	0.032 (4)	0.009 (4)	0.004 (3)	0.004 (3)
C113	0.056 (5)	0.059 (6)	0.047 (5)	0.016 (5)	−0.018 (4)	−0.007 (5)
C114	0.060 (6)	0.064 (7)	0.054 (6)	0.008 (5)	−0.009 (5)	−0.024 (5)
C115	0.049 (5)	0.039 (5)	0.039 (5)	0.015 (4)	0.008 (4)	0.001 (4)
C116	0.034 (4)	0.035 (5)	0.046 (5)	0.004 (3)	−0.005 (3)	−0.003 (4)
C117	0.037 (4)	0.045 (5)	0.050 (5)	−0.003 (4)	−0.009 (3)	0.002 (4)
C118	0.098 (9)	0.063 (8)	0.072 (8)	0.036 (7)	−0.016 (7)	0.003 (6)
C204	0.033 (4)	0.034 (5)	0.055 (5)	0.008 (3)	−0.003 (3)	−0.011 (4)
C205	0.031 (4)	0.052 (6)	0.029 (4)	−0.002 (3)	−0.002 (3)	−0.006 (4)
C206	0.053 (5)	0.054 (8)	0.040 (8)	0.004 (5)	−0.001 (4)	−0.010 (4)
C207	0.029 (3)	0.032 (4)	0.068 (6)	0.002 (3)	−0.013 (4)	−0.003 (4)
C208	0.051 (5)	0.025 (5)	0.107 (10)	0.007 (4)	−0.017 (5)	−0.015 (6)
C209	0.066 (6)	0.026 (4)	0.111 (9)	−0.017 (4)	−0.031 (8)	0.015 (7)
C210	0.052 (5)	0.041 (6)	0.102 (9)	−0.010 (4)	−0.021 (6)	0.020 (6)
C211	0.046 (4)	0.035 (5)	0.068 (6)	−0.004 (4)	−0.009 (4)	0.022 (4)
C212	0.030 (3)	0.045 (5)	0.031 (4)	−0.003 (3)	0.004 (3)	−0.001 (3)
C213	0.042 (4)	0.058 (6)	0.054 (5)	−0.004 (4)	−0.011 (4)	0.005 (5)
C214	0.053 (5)	0.043 (5)	0.054 (6)	0.005 (4)	−0.019 (4)	0.010 (5)
C215	0.043 (4)	0.044 (6)	0.044 (5)	−0.001 (4)	0.001 (4)	0.000 (4)
C216	0.040 (4)	0.068 (7)	0.033 (4)	−0.011 (5)	−0.006 (4)	−0.005 (4)
C217	0.044 (4)	0.040 (5)	0.034 (4)	−0.002 (4)	−0.005 (3)	0.007 (3)
C218	0.092 (9)	0.042 (7)	0.122 (12)	−0.012 (6)	−0.012 (9)	−0.019 (7)

### fac-Bromidotricarbonyl[4-(4-methoxyphenyl)-2-(pyridin-2-yl)thiazole-κ2N,N']rhenium(I) (polymorph-_b) Geometric parameters (Å, º)

**Table:**

Re1—Br1	2.6315 (8)	C210—C211	1.360 (13)
Re2—Br2	2.6308 (8)	C205—C212	1.463 (12)
Re1—N101	2.186 (7)	C212—C213	1.411 (12)
Re1—N102	2.105 (7)	C213—C214	1.379 (14)
Re2—N201	2.185 (7)	O204—C215	1.361 (11)
Re2—N202	2.157 (8)	C214—C215	1.376 (13)
Re1—C101	1.909 (9)	C215—C216	1.373 (13)
Re1—C102	1.919 (9)	C216—C217	1.369 (14)
Re1—C103	1.932 (8)	C212—C217	1.384 (11)
S1—C104	1.713 (9)	O204—C218	1.411 (14)
N101—C104	1.323 (10)	C106—H106	0.9300
N101—C105	1.390 (12)	C108—H108	0.9300
S1—C106	1.711 (13)	C109—H109	0.9300
C105—C106	1.353 (14)	C110—H110	0.9300
N102—C107	1.398 (12)	C111—H111	0.9300
C104—C107	1.452 (13)	C113—H113	0.9300
C107—C108	1.387 (13)	C114—H114	0.9300
C108—C109	1.377 (18)	C116—H116	0.9300
C109—C110	1.364 (19)	C117—H117	0.9300
N102—C111	1.365 (13)	C118—H18A	0.9600
C110—C111	1.386 (13)	C118—H18B	0.9600
C105—C112	1.503 (12)	C118—H18C	0.9600
C112—C113	1.378 (13)	C218—H18D	0.9600
C113—C114	1.372 (15)	C218—H18E	0.9600
O104—C115	1.361 (12)	C218—H18F	0.9600
C114—C115	1.386 (14)	C206—H206	0.9300
C115—C116	1.373 (13)	C208—H208	0.9300
C116—C117	1.411 (12)	C209—H209	0.9300
C112—C117	1.373 (11)	C210—H210	0.9300
O104—C118	1.456 (13)	C211—H211	0.9300
Re2—C201	1.921 (9)	C213—H213	0.9300
Re2—C202	1.909 (10)	C214—H214	0.9300
Re2—C203	1.909 (8)	C216—H216	0.9300
S2—C204	1.707 (9)	C217—H217	0.9300
N201—C204	1.305 (10)	Re1—N101	2.186 (7)
N201—C205	1.387 (11)	Re1—N102	2.106 (7)
S2—C206	1.716 (11)	Re2—N201	2.185 (7)
C205—C206	1.357 (14)	Re2—N202	2.157 (8)
N202—C207	1.317 (14)	C101—O101	1.153 (11)
C204—C207	1.460 (13)	C102—O102	1.149 (10)
C207—C208	1.409 (12)	C103—O103	1.123 (10)
C208—C209	1.374 (19)	C201—O201	1.137 (11)
C209—C210	1.390 (19)	C202—O202	1.162 (11)
N202—C211	1.394 (13)	C203—O203	1.149 (9)
			
C101—Re1—C102	89.4 (4)	N102—C111—H111	116.5
C101—Re1—C103	90.0 (4)	C110—C111—H111	116.5
C102—Re1—C103	91.4 (3)	C117—C112—C113	118.5 (8)
C101—Re1—N102	94.3 (4)	C117—C112—C105	119.6 (8)
C102—Re1—N102	171.4 (3)	C113—C112—C105	121.9 (8)
C103—Re1—N102	96.4 (3)	C114—C113—C112	121.5 (9)
C101—Re1—N101	169.4 (3)	C114—C113—H113	119.3
C102—Re1—N101	100.3 (3)	C112—C113—H113	119.3
C103—Re1—N101	94.1 (3)	C113—C114—C115	120.2 (10)
N102—Re1—N101	75.6 (3)	C113—C114—H114	119.9
C101—Re1—Br1	92.5 (3)	C115—C114—H114	119.9
C102—Re1—Br1	88.9 (2)	O104—C115—C116	124.9 (8)
C103—Re1—Br1	177.5 (2)	O104—C115—C114	115.5 (9)
N102—Re1—Br1	83.20 (19)	C116—C115—C114	119.6 (9)
N101—Re1—Br1	83.40 (16)	C115—C116—C117	119.4 (8)
C202—Re2—C203	89.5 (3)	C115—C116—H116	120.3
C202—Re2—C201	88.1 (4)	C117—C116—H116	120.3
C203—Re2—C201	89.1 (4)	C112—C117—C116	120.8 (8)
C202—Re2—N202	172.8 (4)	C112—C117—H117	119.6
C203—Re2—N202	96.1 (3)	C116—C117—H117	119.6
C201—Re2—N202	96.6 (4)	O104—C118—H18A	109.5
C202—Re2—N201	101.1 (3)	O104—C118—H18B	109.5
C203—Re2—N201	94.8 (3)	H18A—C118—H18B	109.5
C201—Re2—N201	170.0 (3)	O104—C118—H18C	109.5
N202—Re2—N201	73.9 (4)	H18A—C118—H18C	109.5
C202—Re2—Br2	89.3 (2)	H18B—C118—H18C	109.5
C203—Re2—Br2	177.5 (3)	N201—C204—C207	119.5 (8)
C201—Re2—Br2	93.0 (3)	N201—C204—S2	114.2 (7)
N202—Re2—Br2	85.0 (2)	C207—C204—S2	126.2 (7)
N201—Re2—Br2	83.38 (15)	C206—C205—N201	111.4 (9)
O101—C101—Re1	178.9 (9)	C206—C205—C212	126.3 (9)
O102—C102—Re1	176.8 (7)	N201—C205—C212	122.1 (7)
O103—C103—Re1	175.6 (7)	C205—C206—S2	112.6 (9)
O201—C201—Re2	176.2 (9)	C205—C206—H206	123.7
O202—C202—Re2	176.9 (7)	S2—C206—H206	123.7
O203—C203—Re2	178.8 (8)	N202—C207—C208	123.5 (10)
C115—O104—C118	117.1 (8)	N202—C207—C204	112.5 (8)
C215—O204—C218	117.7 (9)	C208—C207—C204	124.1 (10)
C106—S1—C104	90.1 (5)	C209—C208—C207	118.7 (12)
C204—S2—C206	88.7 (5)	C209—C208—H208	120.7
C104—N101—C105	111.8 (8)	C207—C208—H208	120.7
C104—N101—Re1	113.6 (6)	C208—C209—C210	119.4 (9)
C105—N101—Re1	133.2 (6)	C208—C209—H209	120.3
C111—N102—C107	111.9 (8)	C210—C209—H209	120.3
C111—N102—Re1	129.0 (7)	C211—C210—C209	118.4 (10)
C107—N102—Re1	118.9 (6)	C211—C210—H210	120.8
C204—N201—C205	113.0 (7)	C209—C210—H210	120.8
C204—N201—Re2	114.5 (6)	C210—C211—N202	123.8 (11)
C205—N201—Re2	131.7 (6)	C210—C211—H211	118.1
C207—N202—C211	116.2 (8)	N202—C211—H211	118.1
C207—N202—Re2	119.1 (8)	C217—C212—C213	117.8 (8)
C211—N202—Re2	124.5 (8)	C217—C212—C205	120.5 (7)
N101—C104—C107	120.2 (8)	C213—C212—C205	121.7 (8)
N101—C104—S1	113.4 (7)	C214—C213—C212	119.2 (8)
C107—C104—S1	126.4 (7)	C214—C213—H213	120.4
C106—C105—N101	113.6 (9)	C212—C213—H213	120.4
C106—C105—C112	127.1 (9)	C215—C214—C213	122.0 (9)
N101—C105—C112	119.2 (7)	C215—C214—H214	119.0
C105—C106—S1	111.1 (10)	C213—C214—H214	119.0
C105—C106—H106	124.5	O204—C215—C216	125.9 (8)
S1—C106—H106	124.5	O204—C215—C214	115.5 (8)
C108—C107—N102	124.5 (10)	C216—C215—C214	118.6 (9)
C108—C107—C104	124.6 (10)	C217—C216—C215	120.6 (8)
N102—C107—C104	110.9 (7)	C217—C216—H216	119.7
C109—C108—C107	118.7 (12)	C215—C216—H216	119.7
C109—C108—H108	120.6	C216—C217—C212	121.7 (8)
C107—C108—H108	120.6	C216—C217—H217	119.1
C110—C109—C108	120.3 (10)	C212—C217—H217	119.1
C110—C109—H109	119.9	O204—C218—H18D	109.5
C108—C109—H109	119.9	O204—C218—H18E	109.5
C109—C110—C111	117.4 (11)	H18D—C218—H18E	109.5
C109—C110—H110	121.3	O204—C218—H18F	109.5
C111—C110—H110	121.3	H18D—C218—H18F	109.5
N102—C111—C110	127.1 (11)	H18E—C218—H18F	109.5
			
C105—N101—C104—C107	−178.7 (7)	C205—N201—C204—C207	−179.3 (6)
Re1—N101—C104—C107	−10.5 (9)	Re2—N201—C204—C207	−8.3 (9)
C105—N101—C104—S1	−1.0 (8)	C205—N201—C204—S2	−1.1 (8)
Re1—N101—C104—S1	167.2 (4)	Re2—N201—C204—S2	169.8 (3)
C106—S1—C104—N101	−0.2 (6)	C206—S2—C204—N201	0.2 (6)
C106—S1—C104—C107	177.3 (8)	C206—S2—C204—C207	178.2 (7)
C104—N101—C105—C106	2.2 (10)	C204—N201—C205—C206	1.8 (9)
Re1—N101—C105—C106	−163.0 (6)	Re2—N201—C205—C206	−167.2 (5)
C104—N101—C105—C112	−174.1 (7)	C204—N201—C205—C212	−174.0 (7)
Re1—N101—C105—C112	20.8 (11)	Re2—N201—C205—C212	17.1 (10)
N101—C105—C106—S1	−2.3 (10)	N201—C205—C206—S2	−1.6 (9)
C112—C105—C106—S1	173.6 (7)	C212—C205—C206—S2	173.9 (6)
C104—S1—C106—C105	1.4 (7)	C204—S2—C206—C205	0.8 (7)
C111—N102—C107—C108	2.8 (12)	C211—N202—C207—C208	−3.5 (12)
Re1—N102—C107—C108	177.6 (7)	Re2—N202—C207—C208	−179.3 (6)
C111—N102—C107—C104	−179.6 (6)	C211—N202—C207—C204	176.6 (6)
Re1—N102—C107—C104	−4.8 (9)	Re2—N202—C207—C204	0.8 (9)
N101—C104—C107—C108	−172.2 (8)	N201—C204—C207—N202	5.2 (10)
S1—C104—C107—C108	10.5 (13)	S2—C204—C207—N202	−172.7 (6)
N101—C104—C107—N102	10.2 (10)	N201—C204—C207—C208	−174.8 (8)
S1—C104—C107—N102	−167.1 (6)	S2—C204—C207—C208	7.3 (12)
N102—C107—C108—C109	−1.4 (14)	N202—C207—C208—C209	1.3 (14)
C104—C107—C108—C109	−178.6 (9)	C204—C207—C208—C209	−178.8 (8)
C107—C108—C109—C110	1.2 (16)	C207—C208—C209—C210	1.4 (14)
C108—C109—C110—C111	−2.6 (16)	C208—C209—C210—C211	−1.5 (14)
C107—N102—C111—C110	−4.5 (12)	C209—C210—C211—N202	−0.9 (14)
Re1—N102—C111—C110	−178.7 (7)	C207—N202—C211—C210	3.3 (12)
C109—C110—C111—N102	4.6 (15)	Re2—N202—C211—C210	178.9 (7)
C106—C105—C112—C117	−114.7 (10)	C206—C205—C212—C217	−113.9 (10)
N101—C105—C112—C117	61.0 (10)	N201—C205—C212—C217	61.1 (10)
C106—C105—C112—C113	63.7 (13)	C206—C205—C212—C213	66.0 (11)
N101—C105—C112—C113	−120.6 (9)	N201—C205—C212—C213	−119.0 (9)
C117—C112—C113—C114	−2.7 (15)	C217—C212—C213—C214	0.7 (13)
C105—C112—C113—C114	178.9 (9)	C205—C212—C213—C214	−179.3 (8)
C112—C113—C114—C115	−0.3 (17)	C212—C213—C214—C215	0.9 (16)
C118—O104—C115—C116	1.9 (14)	C218—O204—C215—C216	2.4 (15)
C118—O104—C115—C114	−179.0 (10)	C218—O204—C215—C214	−176.7 (11)
C113—C114—C115—O104	−176.6 (10)	C213—C214—C215—O204	178.7 (9)
C113—C114—C115—C116	2.5 (16)	C213—C214—C215—C216	−0.5 (16)
O104—C115—C116—C117	177.5 (8)	O204—C215—C216—C217	179.3 (8)
C114—C115—C116—C117	−1.5 (14)	C214—C215—C216—C217	−1.5 (14)
C113—C112—C117—C116	3.6 (13)	C215—C216—C217—C212	3.2 (13)
C105—C112—C117—C116	−177.9 (8)	C213—C212—C217—C216	−2.7 (12)
C115—C116—C117—C112	−1.6 (13)	C205—C212—C217—C216	177.2 (8)

### fac-Bromidotricarbonyl[4-(4-methoxyphenyl)-2-(pyridin-2-yl)thiazole-κ2N,N']rhenium(I) (polymorph-_b) Hydrogen-bond geometry (Å, º)

**Table:**

*D*—H···*A*	*D*—H	H···*A*	*D*···*A*	*D*—H···*A*
C108—H108···O104^i^	0.93	2.66	3.435 (13)	141
C109—H109···O102^i^	0.93	2.50	3.397 (14)	161
C208—H208···O204^i^	0.93	2.66	3.442 (13)	142
C209—H209···O202^i^	0.93	2.43	3.337 (13)	166
C210—H210···O101	0.93	2.72	3.110 (12)	106
C214—H214···O104^ii^	0.93	2.65	3.365 (12)	135
C117—H117···Br1^iii^	0.93	3.03	3.873 (9)	152
C118—H18*A*···Br1^iv^	0.96	2.94	3.868 (12)	162
C206—H206···Br1^v^	0.93	3.03	3.845 (14)	148
C106—H106···Br2^vi^	0.93	3.06	3.762 (14)	133
C218—H18*D*···Br2^vii^	0.96	2.97	3.865 (12)	155

Symmetry codes: (i) *x*, *y*−1, *z*; (ii) −*x*+1, −*y*+2, *z*−1/2; (iii) *x*−1/2, −*y*+1/2, *z*; (iv) *x*−1/2, −*y*+3/2, *z*; (v) −*x*+3/2, *y*+1/2, *z*−1/2; (vi) −*x*+1, −*y*+1, *z*+1/2; (vii) *x*+1/2, −*y*+5/2, *z*.
